# Effects of Sleep Deprivation by Olfactorily Induced Sexual Arousal Compared to Immobilization Stress and Manual Sleep Deprivation on Neuromessengers and Time Keeping Genes in the Suprachiasmatic Nuclei and Other Cerebral Entities of Syrian Hamsters—An Immunohistochemical Study

**DOI:** 10.3390/ijerph18179169

**Published:** 2021-08-31

**Authors:** Christian Knöchel, Hagen Frickmann, Frank Nürnberger

**Affiliations:** 1 Vitos Clincis of Forensic Psychiatry Eltville, 65346 Eltville, Germany; Christian.knoechel@vitos-rheingau.de; 2 Department of Microbiology and Hospital Hygiene, Bundeswehr Hospital Hamburg, 20359 Hamburg, Germany; hagen_frickmann@hotmail.com or; 3 Institute for Medical Microbiology, Virology and Hygiene, University Medicine Rostock, 18057 Rostock, Germany; 4 Institute for Anatomy II, Goethe-Universität Frankfurt am Main, 60590 Frankfurt am Main, Germany

**Keywords:** sexual arousal, sleep deprivation, Syrian hamster, suprachiasmatic nucleus, aphrodisin, pheromone, neuropeptide, neurotransmitter

## Abstract

We investigated the effects of sexual arousal induced by olfactory stimuli on the expression of neuromodulators, neurotransmitters and sexual steroid receptors in the suprachiasmatic nucleus (SCN, the circadian pacemaker of mammals) and other cerebral entities of Syrian hamsters (*Mesocricetus auratus*) compared to manual sleep deprivation and immobilization stress. The hamsters kept under a 12:12 hours (h) light:dark cycle were deprived of sleep by sexual stimulation, gentle manual handling or immobilization stress for 1 h at the beginning of the light phase and subsequently sacrificed at zeitgeber time 01:00, respectively; for comparison, hamsters were manually sleep deprived for 6 or 20 h or sacrificed after completing a full sleep phase. As demonstrated by immunohistochemistry, apart from various alterations after manual sleep deprivation, sexual stimulation caused down-regulation of arginine-vasopressin (AVP), vasointestinal peptide (VIP), serotonin (5-HT), substance P (SP), and met-enkephalin (ME) in the SCN. Somatostatin (SOM) was diminished in the medial periventricular nucleus (MPVN). In contrast, an increase in AVP was observed in the PVN, that of oxytocin (OXY) in the supraoptic nucleus (SON), of tyrosine-hydroxylase (TH) in the infundibular nucleus (IN), and dopamine beta-hydroxylase (DBH) in the A7 neuron population of the brain stem (A7), respectively. Testosterone in plasma was increased. The results indicate that sexual arousal extensively influences the neuropeptide systems of the SCN, suggesting an involvement of the SCN in reproductive behavior.

## 1. Introduction

Different modulator and transmitter systems of various hypothalamic nuclei are involved in sexual control: arginine-vasopressin (AVP), dopamine, oxytocin (OXY), and substance P (SP) next to sexual steroids facilitate sexual behavior [1,2,3,4,5], whereas neuropeptide Y (NP Y), somatostatin (SOM), endogenous opioids such as met-enkephalin (ME), serotonin (5-HT = 5-hydroxy-tryptamin) and gamma-amino butyric acid (GABA) express inhibitory influences on sexual activity [6,7,8,9,10]. Most of these messengers are observed in the suprachiasmatic nuclei (SCN), which are located in the anterior hypothalamus. Although these nuclei predominantly represent the master circadian pacemakers in mammals, which rule the cycle of sleep and alertness [11], the SCN may also control particular aspects of sexual behavior, which is further supported by the occurrence of GRP and VIP within this entity. The latter peptides are involved, at least, in the peripheral control of reproductive organs [12,13], although some reports on central nervous involvement are available [14].

The main output projections of the SCN terminate in the magnocellular paraventricular nucleus (PVN), which conveys biorhythmic cues to the peripheral organism via the autonomic nervous system and the endocrine axis [15]. Furthermore, the SCN regulates the neurons of the supraoptic nucleus (SON), the main source of diurnally-rhythmically released OXY [16]. In addition, the SCN is influenced by somatostatinergic afferences from the medial periventricular nucleus (MPVN) [17] and serotoninergic afferences from the dorsal raphe nucleus [18,19], the latter itself being influenced by norepinephrinergic afferences from the brain stem [20]. Norepinephrinergic afferences are known to directly or indirectly influence the circadian regulation of arousal [21].

To elucidate the stability of the diurnal rhythmic alterations of the above-mentioned messenger systems, we experimentally affected the natural diurnal rhythm by sleep deprivation at the beginning of the main sleep phase of Syrian hamsters which starts in the early light phase. We studied the effect of sleep deprivation [22] on selected neuropeptide and neurotransmitter systems in comparison to animals that could sleep ad libitum. In particular, we compared such effects after gentle manually or stress-induced sleep deprivation with effects of a particular kind of reproductive sleep deprivation, i.e., the stimulation of sexual behavior by pheromones and the presence of females.

This type of sexual sleep deprivation in male Syrian hamsters was induced by showing female hamsters in adjoining cage compartments combined with exposition to the pheromone aphrodisin, a soluble glycoprotein of the vagina of hamsters proven to stimulate mating behavior in male hamsters. Dimeric recombinant aphrodisin molecules bind to dimethyl-disulfide which acts at pheromone receptor neurons in the basal compartment of the vomeronasal epithelium axonally connected with the caudal region of the accessory olfactory bulb [23,24,25].

In order to distinguish the effects of sexually driven sleep deprivation from that of alternative waking signals and the pheromone effects alone, we compared the sexually stimulated group with additional control groups, one being deprived of sleep for the same interval and during the same diurnal phase by gentle manual handling, another one by pheromone exposition alone, and a third one by the aversive stimulus of immobilization stress, respectively. The scientific goals were the induction of sexual arousal and the observation of specific patterns on precisely localized neurotransmitter and neuropeptide reactivity.

The assessment was focused on sleep deprivation by sexual arousal rather than on sexual arousal solely due to the assumption that sexual arousal-induced effects may be more obvious during diurnal phases of tiredness or inactivity rather than during periods of natural alertness. Therefore, the experiments were performed during the resting period and not during the typical mating period usually occurring during the night phase.

## 2. Materials and Methods

### 2.1. Laboratory Animals

Fifty-three sexually naïve adult male Syrian golden hamsters (*Mesocricetus auratus*) were obtained from Charles River (Sulzfeld, Germany) at the age of 3 weeks and kept in groups of five animals (litter mates) per cage. Free access to food (rat pellets) and water was guaranteed. Constant temperature of 20 °C and a day-night-periodicity of 12 h light and 12 h darkness were provided for at least 8 weeks (lights on 07:00 = zeitgeber time [ZT] 00:00). This light regimen was chosen since the hamsters develop their sexual maturity under those conditions; their natural circadian period length τ is approximately 23 h. All hamsters were tamed to avoid unintended stress-specific effects. The taming was achieved by frequent manual interactions, i.e., lifting the animals out of the cages, soft stroking, and gentle touching. Principles of laboratory animal care and specific national laws were followed in line with the German standards when the experiments were performed between 2003 and 2005. In detail, all experiments were in accordance with a protocol approved by the Policy on Ethics, as approved by the Society for Neuroscience, and which was consistent with Federal guidelines and the European Communities Council Directive.

### 2.2. Pre-Tests Regarding the Animals’ Behavior

In order to verify the diurnal sleep cycle and the main diurnal sleep phase of the Syrian hamsters used in the present investigation, their sleeping behavior was observed during the entire diurnal cycle prior to the experiments described below. The pre-test studies also included the observation of the activity patterns of the hamsters exposed to olfactory stimulation with pheromone and optical stimulation with female hamsters during the pre-noon light phase, i.e., the main diurnal sleep phase. Furthermore, the behavior of the hamsters during prolonged sleep deprivation (6 h, 20 h) was observed. We decided not to show activity or sleep profiles, since our animals did behave according to the descriptions published in the recent literature, cf., e.g., [26].

### 2.3. Sexual Stimulation, Immobilization Stress, and Gentle Manual Handling

The experiments were performed during the 12th week of life of the golden hamsters at a time when the animals were sexually mature but still naïve. Accordingly, social stress and obesity did not occur at that early stage of maturity. The cages—each containing four or five male hamsters which were kept together and knew each other (as litter mates)—were assigned to the experimental groups. All experiments were performed under conditions of bright light at the beginning of the light-phase.

For the pre-tests on the basic effects of sleep deprivation by gentle manual handling, a control group was defined which was allowed to sleep and sacrificed at zeitgeber time 05.00 at the end of their natural sleeping period. A short-term sleep deprivation group, which was sacrificed at the same time, was kept awake for the whole sleeping period by gentle manual handling starting 1 h prior to the onset of the light phase, resulting in a total of 6 h sleep deprivation. Furthermore, a prolonged sleep deprivation group, for which gentle manual handling started at zeitgeber time 09.00 of the day before, resulting in 20 h sleep deprivation before the animals were sacrificed at zeitgeber time 05.00, was added.

Because the sexual stimulation experiment (as described in detail below) failed to keep the animals awake for the whole sleeping period, the sacrificing time for the comparison of sleep deprivation due to gentle manual handling, exposition to pheromones alone, exposition to pheromones and females, and immobilization stress, respectively, was set at zeitgeber time 01.00.

Accordingly, the sexual stimulation group consisted of animals that were deprived of sleep at the beginning of their main sleeping period starting at ZT 00:00 by sexual stimulation for 1 h. The pheromone used for the olfactory stimulation consisted of a solution containing ~35 mg/L recombinant aphrodisin produced in yeast cells [27]. This stock solution, which was supplemented with 1% yeast extract, 2% peptones, 100 mM/L potassium phosphate, 1.34% yeast mitogen base with ammonium sulfate and 0.5% methanol, was a donation of Privatdozent Dr. Hans-Jürgen Mägert and Dr. Michael Walden. Just before application in the experiment, 10 mL of dimethyl disulfide (DMDS 1 g/mL) was added to 500 mL of the aphrodisin-containing solution.

- Group 1, sexual stimulation with ahrodisin and females: A hamster cage (Makrolon cage type III) was divided in its centre by a double layer of flywire. Two cellulose balls soaked with the aphrodisin-DMDS-mixture were placed under the bedding of one area, five adult female hamsters (age ≥ 3 months) in the other area. The female Syrian hamsters (purchased from Charles River, Sulzfeld, Germany) were kept under the same conditions as the males but completely separated in another room. According to literature, female hamsters are sexually sensible for one day each 4 to 7 days at median daily temperature of 15 °C [28]. When the experiment started, five male hamsters were placed in the pheromone-containing compartment. They were able to see, hear and smell the females through the wire-netting without being able to physically contact them. The aphrodisin-DMDS-mix was additionally applied to the cage with a vaporizer. This experiment was performed twice, resulting in materials from 10 hamsters.
- Group 2, sexual stimulation with aphrodisin solely: The experimental setting of the pheromone-only group consisting of five hamsters was the same as described for group 1 with the exception that no female animals were present.
- Group 3, manual sleep deprivation: The manual sleep deprivation group was deprived of sleep by gentle manual handling whenever the hamsters tended to fall asleep during the same period of the diurnal phase starting at ZT 00:00. This group was subdivided into 10 animals sleep deprived for 1 h (ZT 00:00 to ZT 01:00, later referred to as group 3a (group 3a represents the principle control group for groups 1, 2 and 4)), five hamsters sleep deprived for 6 h (ZT 23:00 to ZT 05:00, later referred to as group 3b) and nine animals sleep-deprived for 20 h (ZT 09:00 to ZT 05:00 of the following day, later referred to as group 3c). (Groups 3b and 3c were introduced for deciphering possible changes after different durations of sleep deprivation).
- Group 4, sleep deprivation by stress: Finally, the last comparison group was exposed to immobilization stress by nearly complete immobilization caused by wrapping them with extra strong scotch tape. The head remained unwrapped. Thus, ventilation was still guaranteed. All comparison groups for the sexual stimulation group were also sacrificed at zeitgeber time 01.00.
- Group 5, sleep control: Nine hamsters were sacrificed after undisturbed sleep for 5 h at ZT 05:00. A further group studied after 1 h of sleep was not used, since there are no significant changes in comparison to 5 h of sleep, however, the experimental setup to prevent the effects of disturbed sleep after 1 h is rather complicated as shown in [29].

### 2.4. Sample Acquisition and Preparation

All hamsters were sacrificed by decapitation, either at ZT 01.00 to 01.15 or at ZT 05:00 to 05:15, depending on the experimental group. Blood was collected via a heparinized funnel (Liquemin^®^, Hoffmann-La Roche AG, Basel, Switzerland) in EDTA tubes and centrifuged; plasma was separated and frozen at −20 °C. All brains were quickly dissected out of the skull, the hemispheres of the telencephalon were removed and the remaining brain tissue was immersion-fixed in Zamboni’s solution (4% paraformaldehyde plus 15% saturated picric acid in 0.1 M phosphate buffer) [30] for 2 days. (Decapitation was chosen because of the blood collection; based on our fast processing of dissection and fixation, the immunoreactivity is equivalent to perfusion.) After fixation, the brains were submerged in a graded concentration series of sucrose for cryoprotection (1 day (d) 10%, 2 d 20%, 2 d 30% in 0.1 M phosphate buffer). Following sucrose infiltration, the tissue was quickly frozen and serial coronal cryostat sections (20 µm) were mounted on double-gelatinized glass slides.

### 2.5. Immunohistochemistry

Sections were processed by immunohistochemistry using the biotin–avidin peroxidase technique (VECTASTAIN Elite ABC, Vectorlabs, Burlingame, CA, USA) and 3.3′-diaminobenzidine (DAB) visualization as described [31,32] with various anti-sera (Table 1). Thereby, not all analyses were performed for all experimental groups, depending on the availability of sufficient numbers of brain slices and antibody volumes. In such cases, respective parameters were assessed only for the experimental groups for which sufficient sample materials were available.

The antibodies used for the immunohistochemical analyses were assessed for specificity in our laboratories of the Institute of Anatomy of the University of Frankfurt/Main, Germany. In particular, cross-reactivity to related peptide sequences was excluded, antibodies after preincubation with specific antigen did not stain, and replacement of individual reaction steps by buffers prevented immunostaining.

The semiquantitative analysis of the immunostained sections was based on planimetric methods as previously described for the SCN [29,31]. In all other nuclei, the assessed areas were placed within the core of the various nuclei without crossing their boundaries. The area of the specifically stained structures (perikarya and fibers) was calculated as percent of the representative sample area of the brain nucleus (mean values). The outlines of the three brain nuclei studied could be easily recognized by their specific immunohistochemical staining patterns and by their cytoarchitecture as verified by microscopic observation applying the Nomarski technique (Zeiss Axioplan, Oberkochen, Germany).

The microscope (Fotomicroscope III, Zeiss, Göttingen, Germany) was connected to the computer via a video camera and an A/D image converter. In order to have reproducible illumination conditions at the microscope, the Köhler illumination principle, a 10× photo eyepiece, the 1.25× “optoview” setting and the objectives 25× (plan 25/0.45; 160/0.17; Carl Zeiss 5130955; Göttingen, Germany) and 40× (plan 40/0.65; 160/0.17; Zeiss West Germany 460710-9904; Göttingen, Germany) were used. All internal filters were removed and the field diaphragm was set to the field of view size. In this way, and due to a lamp voltage setting that was the same for all analysis sessions, reproducible illumination was ensured. For better contrast of the diaminobenzidine staining, an illumination wavelength of 520 nm (green light) was set on an interference gradient filter.

The following evaluations were made with the 25× objective: AVP, VIP, GRP, GAD65/67, NPY, OXY in the SON and PVN, AR in the PVN, OR in the PVN, TH, DBH, 5-HT in the dorsalis raphe nuclei. The following evaluations were made with the 40× objective: SP, ME, SOM, AR in the medial pre-optic area as well as in the medial periventricular nucleus and OR in the medial preoptic area. Depending on the selected objective and the resulting size of the sample area, different relative proportions of the stained area resulted. For example, a stained fiber or a stained perikaryon represented a larger area relative to the sample area when using the 40× objective than using the 25× objective.

Using the computer-assisted image analysis system VIDAS (Vidas 2.1^®^; Kontron, Eiching, Germany), a representative rectangular area of constant size was selected within each stained nuclear area. Within this reference area, the total area of immunostained cells and fibers was measured and expressed as a percentage of the sample area. Thereby, six serial sections in 100 µm distances per nucleus were used for evaluation, showing comparable sections of the respective nuclear area. The threshold of positive immunostaining was determined using a densitometric paradigm: After computer contrast enhancement, the absorbance of specific immunostaining was interactively selected. Non-specific artifact staining was interactively deleted and thus not recorded.

The photographic documentation was carried out on a microscope “axioscope” (Zeiss, Göttingen, Germany) attached with a digital camera. Representative sections that had previously been evaluated with the VIDAS 2.1^®^ (Kontron, Eiching, Germany)-supported analysis technique were selected for documentation. The digitized images were processed with the Axio Vision 3.1^®^ program from Carl Zeiss Vision GmbH (Zeiss, Göttingen, Germany).

Due to excessive background staining, the serotonergic immunoreactivity in the fibers in the area of the suprachiasmatic nucleus could not be evaluated on the computer. Therefore, since only serotonin-containing nerve fibers but no serotonin-containing perikarya occur in the SCN [33,34], a camera-lucida technique [35,36] was used. For this purpose, the serotonergic fibers in the ventromedial part of the suprachiasmatic nucleus were visualized in representative sections at 400× magnification under the microscope. Using deflection prisms (drawing tube for “Axioplan” microscopes, Zeiss, Göttingen, Germany), the image was projected onto a white sheet of standard DIN A4 copy paper, where the fiber courses were traced using a black fiber pen (Stabilo^®^-True Marker, STABILO International Gmbh, Heroldsberg, Germany) with a minimum diameter of 0.4 mm. Again, six drawings were made per nucleus. The sheets were scanned with a “Network HighScan professional^®^” (MediaMarkt, Ingolstadt, Germany) scanner in greyscale at 72 dpi (dots per inch) and saved as “bmp” formats with 567 × 796 pixels each. The number of black pixels was calculated and indicated for each image using the program Adope Photoshop 7.0^®^ (Adope Systems Engineering, Hamburg, Germany).

### 2.6. Blood Analyses

Blood levels of arginine-vasopressin and testosterone were analyzed by use of radio-immunoassay (AVP, Vasopression RIA RK-AR1, Bühlmann Laboratories, Schönenbuch, Switzerland) or chemiluminescence-immunoassay (testosterone, Advia Centaur Testosteron Test, Advia Centaur, Bayer Corporation, Leverkusen, Germany) techniques as commissioned work by Biocontrol (Ingelheim, Germany).

### 2.7. Statistics

Statistical assessment was based upon many neurobiological sections of few individuals. About six sections per animal, i.e., 12 sections of bilaterally located nuclei, in five animals = 60 samples per experimental group were analyzed, while the number of assessed animals was kept small for ethical reasons. The anterior–posterior distance between each section was 100 µm. Mean values of the results of those serial sections were included in the assessments. However, due to the low number of animals per experimental group, only non-parametric methods were applied, i.e., Whitney–Mann U testing if only two experimental groups were compared and Kruskal–Wallis testing as well as subsequent post-hoc testing with Dunn–Bonferroni tests in case of more than two experimental groups. Statistical assessments were performed applying the software GraphPad Instat version 3.06 (GraphPad Software Inc., San Diego, CA, USA). Significance was accepted at *p* < 0.05.

## 3. Results

### 3.1. Behavioral Changes

The observations during the pre-tests verified that the diurnal sleep phase started at the end of the dark period shortly before ZT 00:00. This main diurnal sleep phase continued for 4–6 h, obvious arousal reactions were not observed. Accordingly, the pretests on the comparison of undisturbed sleep with short-term manual sleep deprivation for 6 h and intermediate-term manual sleep deprivation for 20 h were timed to end at ZT 05.00.

The pretests on sexual stimulation with exposition to pheromone and female hamsters led to a tremendous increase in physical activity and arousal of the male hamsters at ZT 00:00 when the animals usually started sleeping. Until ZT 01:30, the hamsters intensively tried to cross the barrier to contact the females. At ZT 02:00, two males fell asleep, at ZT 02:30 only one male still tried to cross the barrier, at ZT 3:30 all-male hamsters were sleeping. Similar, slightly less pronounced activity was observed in the pheromone-only group.

According to the observed time span of maximum courtship behavior, the sacrifice of the groups of hamsters for the comparison of sleep deprivation due to manual handling, sexual stimulation by pheromones and females, pheromone stimulation-only and immobilization stress was set at 60 min after the start of the experiment. Therefore, the animals were sacrificed at ZT 01.00. The immobilized group showed high levels of alertness during all 60 min of immobilization, tried to perform escape maneuvers and to bite until decapitation after the same interval. The hamsters that were deprived of sleep by gentle manual handling showed signs of tiredness such as attempts of achieving curled-up sleeping positions until decapitation.

### 3.2. Changes of Immunoreactivity to Neuromessengers, Time Keeping Proteins and Steroid Receptors in Cerebral Entities as Well as Their Concentrations in Blood

**Figure 1:**
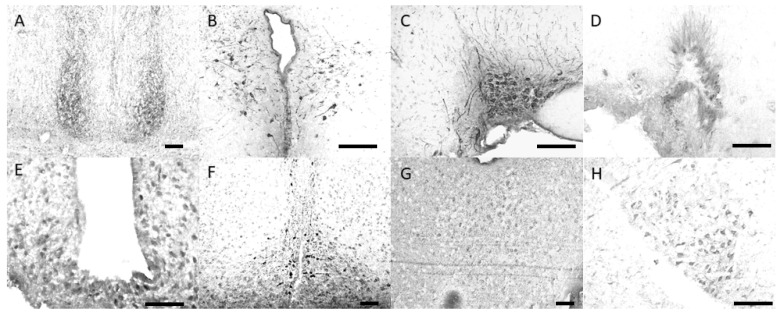
Example microphotographs of cerebral entities of male Syrian hamsters analyzed in the present investigation. (**A**) suprachiasmatic nucleus (anti-gastrin releasing peptide staining), (**B**) paraventricular nucleus (anti-arginine-vasopressin staining), (**C**) supraoptic nucleus (anti-arginine vasopressin staining), (**D**) medial preoptic area (anti-androgen receptor staining), (**E**) medial periventricular nucleus (anti-somatostatin staining), (**F**) infundibular nucleus (anti-tyrosine hydroxylase staining), (**G**) dorsal raphe nucleus (anti-serotonin staining), (**H**) A7 neuron population (anti-dopamine beta-hydroxylase staining). Scale bar 100 µm.

An overview of the assessed cerebral entities is provided in Figure 1. Immunoreactivity to antisera against arginine-vasopressin (AVP), vasoactive intestinal polypeptide (VIP), gastrin-releasing peptide (GRP) and GAD-65/67 was detected in perikarya and fibers of the suprachiasmatic nucleus (SCN) of golden hamsters while there was immunoreactivity to substance P (SP), serotonin (5-HT), met-enkephalin (ME), and neuropeptide Y (NP-Y) in fibers only.

Both short-term manual sleep deprivation settings, i.e., sleep deprivation for 6 h with assessment at ZT 05.00 (group 3b) and sleep deprivation for 1 h with assessment at ZT 01.00 (group 3a), did not show significantly different results with the exception of higher oxytocin (OXY) immunoreactivity in the paraventricular nucleus (PVN) in the 1-h sleep deprivation group. For all other comparably assessed entities, there was no difference between the two short-term sleep deprivation groups.

Lower OXY levels in the PVN compared to the 1-h manual sleep deprivation group (group 3a) were also seen in the sleeping animals (group 5). In the supraoptic nucleus (SON), in contrast, lower OXY levels were observed in group 2 (pheromone stimulation only) as compared to both the 1-h sleep deprivation group (group 3a) and the sexual-stimulation group 1, respectively.

A highly differentiated pattern was seen for AVP in the different assessed hypothalamic entities as well as in blood. In the SCN, increased AVP immunoreactivity was observed in the 1-h manual sleep deprivation group (group 3a) compared to the sleeping control animals (group 5) as well as to the animals exposed to sexual stimulation (group 1) and immobilization stress (group 4). In the PVN, in contrast, AVP levels of sexually stimulated hamsters (group 1) were increased compared to sleeping controls. In the SON, immobilization stress led to reduced AVP immunoreactivity compared to all three manual handling-induced sleep deprivation settings (group 3), while only the 1-h manual sleep deprivation group 3a showed significantly higher AVP levels compared to group 2 (pheromone only), too. In blood, in contrast, 5-HT was significantly increased in the pheromone-only group 2 compared to the 1-h manual sleep deprivation group 3a.

A decrease in the time-keeping proteins Clock and Period (Per) 2 in the SCN of sleeping animals compared to animals deprived of sleep for 20 h (group 3c) was observed, while such changes were undetectable for Per 1 and cryptochrome (Cry) 2 in the SCN.

In the PVN, immunoreactivity of androgen receptors (AR) and estrogen receptors (OR) were decreased in the sexually stimulated group 1 compared to the 1-h manual sleep deprivation group 3a. In the medial preoptic nucleus (MPOA), in contrast, this decrease was not observable. Although Kruskal-Wallis testing suggested the significance of an overall difference between the groups assessed for AR in the MPOA, the post-hoc analyses indicated no further significance for the comparisons of the different experimental groups.

A similar pattern of reduced immunoreactivity in sexually stimulated hamsters (group 1) compared to hamsters deprived of sleep by gentle handling for 1 h (group 3a) was also observed for somatostatin (SOM) in the medial periventricular nucleus (MPVN).

Focusing on tyrosine-hydroxylase (TH), the guiding enzyme of dopamine synthesis, in the infundibular nucleus (IN), sleeping animals (group 5) showed lower immunoreactivity compared to animals manually deprived of sleep for 1 h (group 3a) and sexually stimulated animals (group 1), respectively, while only sexually stimulated hamsters (group 1) showed more TH compared to immobilized hamsters (group 4) as well. In sleeping hamsters (group 5), dopamine beta-hydroxylase (DBH), the guiding enzyme of epinephrine synthesis, was also decreased in the A7 neuron population of the brainstem compared to the sexual stimulation group 1 and the 20-h manual sleep deprivation group 3c.

Both sexual arousal, achieved by combining pheromone with contact to females (group 1), and short-term manual sleep deprivation for 6 h (group 3b) were associated with elevated levels of testosterone in blood compared to immobilization stress (group 4). Details of the above-mentioned facts are summarized in Table 2.

As shown in Table 3, short-term manual sleep deprivation for 1 h (group 1) led to an increase in the reactivity of most of the assessed messenger systems, while nearly uniform decreases were observed for sleeping (group 5) and immobilized (group 4) hamsters. In contrast, both prolonged sleep deprivation as well as stimulation either by pheromone alone (group 2) or by pheromone and females (group 1) led to a more differentiated response. Thereby, however, it was obvious that the addition of females to sole pheromone stimulation led to a reaction pattern considerably different than in the case of pheromone application alone.

**Figure A1:**
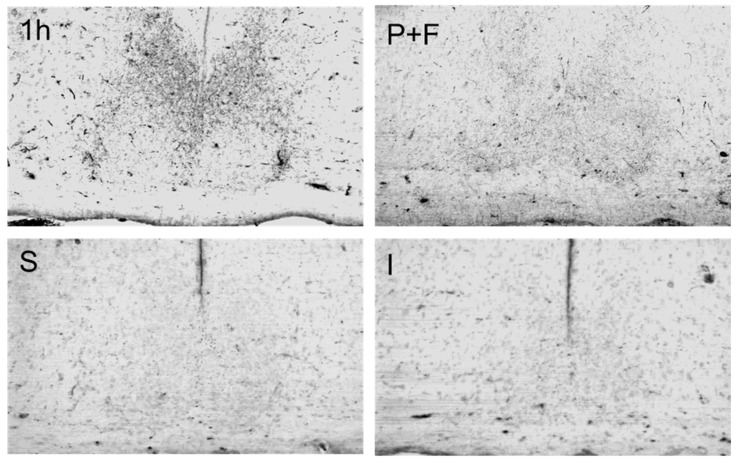
Arginine-vasopressin immunoreactivity in the suprachiasmatic nuclei of Syrian hamsters. Comparison between 1 h manual sleep deprivation (1 h, group 3a), sexual stimulation with pheromone and females (P+F, group 1), sleep (S, group 5) and immobilization stress (I, group 4). Magnification: 50×.

**Figure A2:**
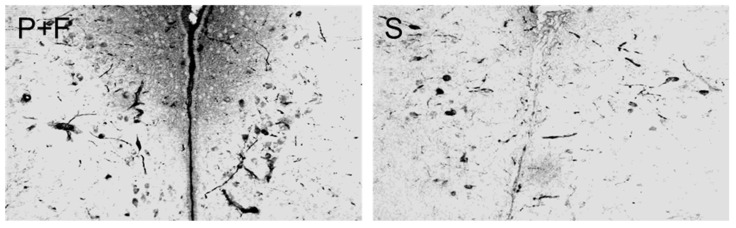
Arginine-vasopressin immunoreactivity in the paraventricular nuclei of Syrian hamsters. Comparison between sexual stimulation with pheromone and females (P+F, group 1) and sleep (S, group 5). Magnification: 50×.

**Figure A3:**
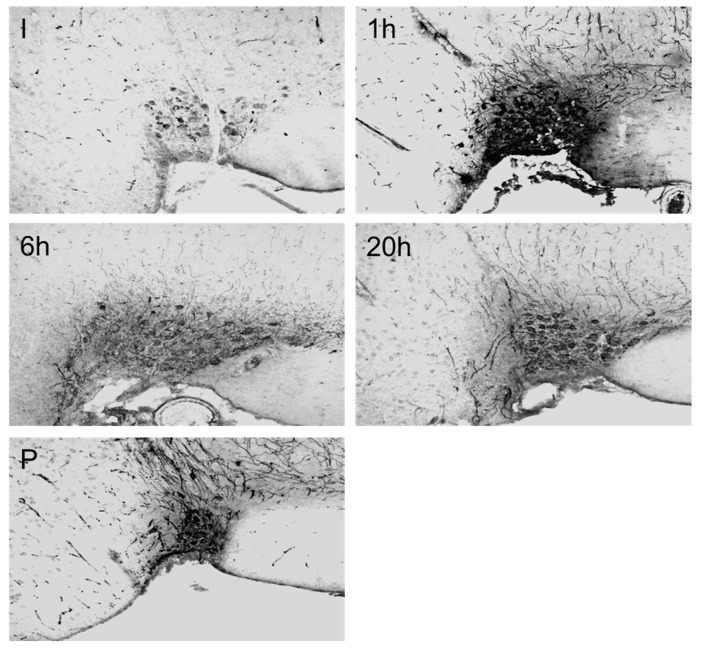
Arginine-vasopressin immunoreactivity in the supraoptic nucleus of Syrian hamsters. Comparison among immobilization stress (I, group 4), 1 h manual sleep deprivation (1 h, group 3a), 6 h manual sleep deprivation (6 h, group 3b), 20 h manual sleep deprivation (20 h, group 3c) and sexual stimulation with pheromone (P, group 2). Magnification: 50×.

**Figure A4:**
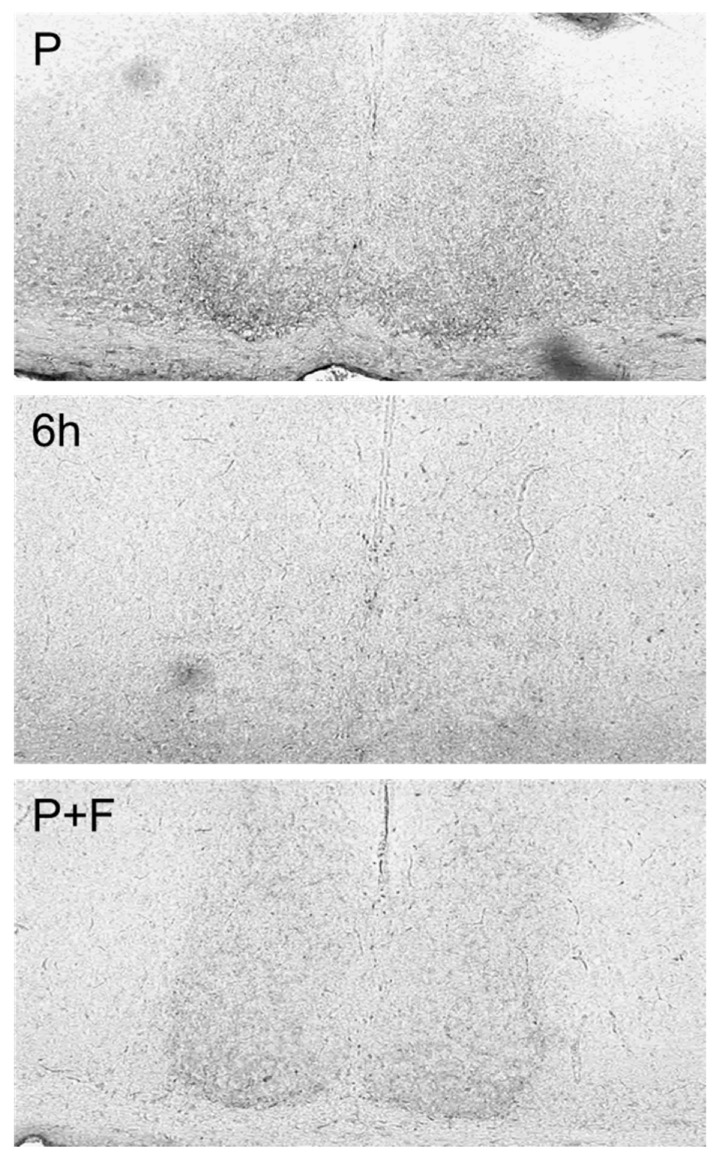
Vasoactive-intestinal peptide immunoreactivity in the suprachiasmatic nuclei of Syrian hamsters. Comparison between sexual stimulation with pheromone (P, group 2), 6 h manual sleep deprivation (6 h, group 3b), and sexual stimulation with pheromone and females (P+F, group 1). Magnification: 50×.

**Figure A5:**
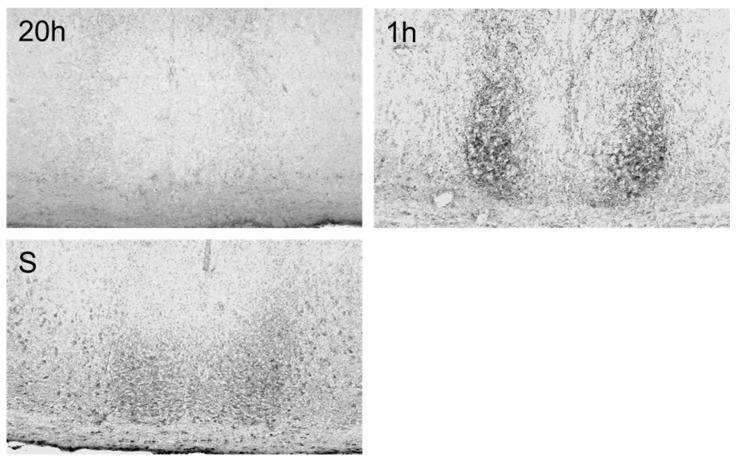
Gastrin releasing peptide immunoreactivity in the suprachiasmatic nuclei of Syrian hamsters. Comparison between 20 h manual sleep deprivation (20 h, group 3c), 1 h manual sleep deprivation (1 h, group 3a) and sleep (S, group 5). Magnification: 50×.

**Figure A6:**
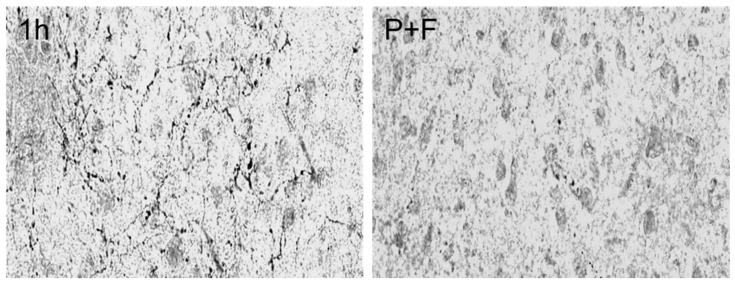
Substance P immunoreactivity in the suprachiasmatic nucleus of Syrian hamsters. Comparison between 1 h manual sleep deprivation (1 h, group 3a) and sexual stimulation with pheromone and females (P+F, group 1). Magnification: 200×.

**Figure A7:**
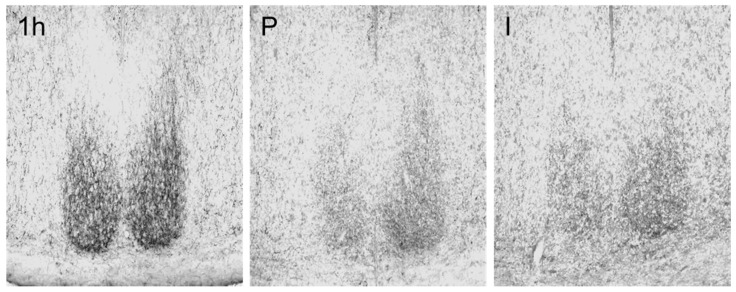
Neuropeptide Y immunoreactivity in the suprachiasmatic nucleus of Syrian hamsters. Comparison between 1 h manual sleep deprivation (1 h, group 3a), sexual stimulation with pheromone (P, group 2), and immobilization stress (I, group 4). Magnification: 40×.

**Figure A8:**
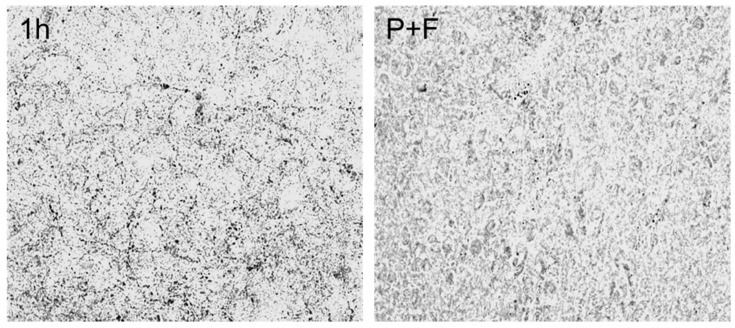
Methionin-enkephalin immunoreactivity in the suprachiasmatic nucleus of Syrian hamsters. Comparison between 1 h manual sleep deprivation (1 h, group 3a) and sexual stimulation with pheromone and females (P+F, group 1). Magnification: 200×.

**Figure A9:**
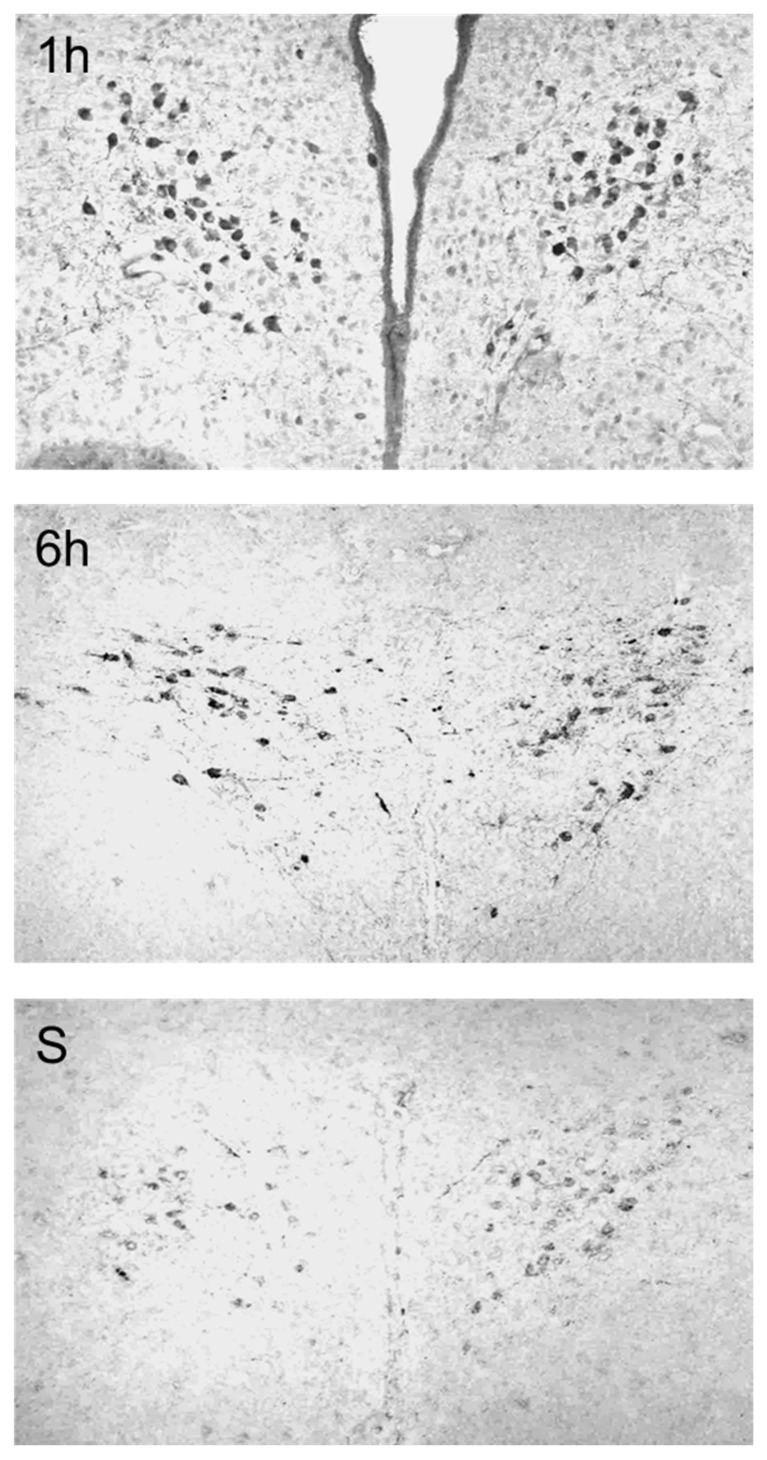
Oxytocin immunoreactivity in the paraventricular nuclei of Syrian hamsters. Comparison among 1 h manual sleep deprivation (1 h, group 3a), 6 h manual sleep deprivation (6 h, group 3b) and sleep (S, group 5). Magnification: 80×.

**Figure A10:**
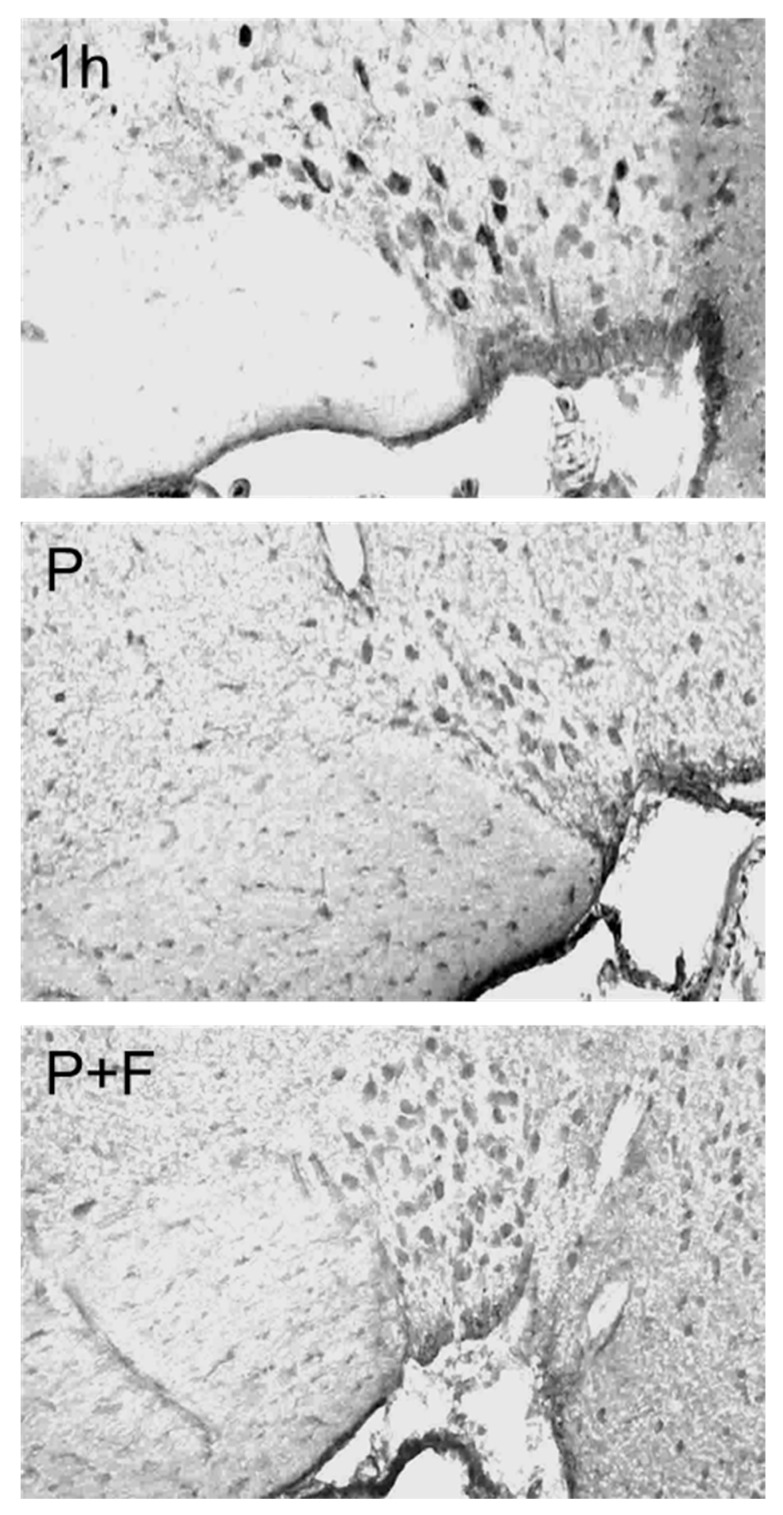
Oxytocin immunoreactivity in the supraoptic nucleus of Syrian hamsters. Comparison between 1 h manual sleep deprivation (1 h, group 3a), sexual stimulation with pheromone (P, group 2), and sexual stimulation with pheromone and females (P+F, group 1). Magnification: 80×.

**Figure A11:**
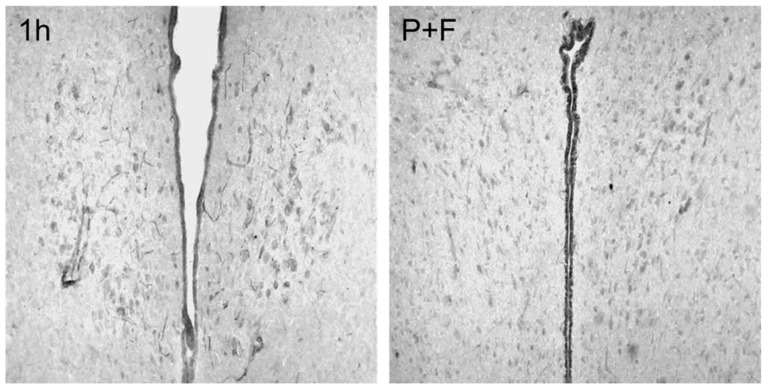
Androgen-receptor immunoreactivity in the paraventricular nuclei of Syrian hamsters. Comparison between 1 h manual sleep deprivation (1 h, group 3a) and sexual stimulation with pheromone and females (P+F, group 1). Magnification: 80×.

**Figure A12:**
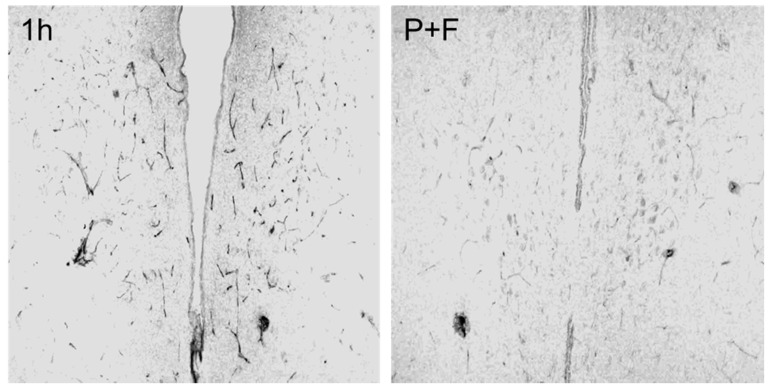
Estrogen-receptor immunoreactivity in the paraventricular nuclei of Syrian hamsters. Comparison between 1 h manual sleep deprivation (1 h, group 3a) and sexual stimulation with pheromone and females (P+F, group 1). Magnification: 50×.

**Figure A13:**
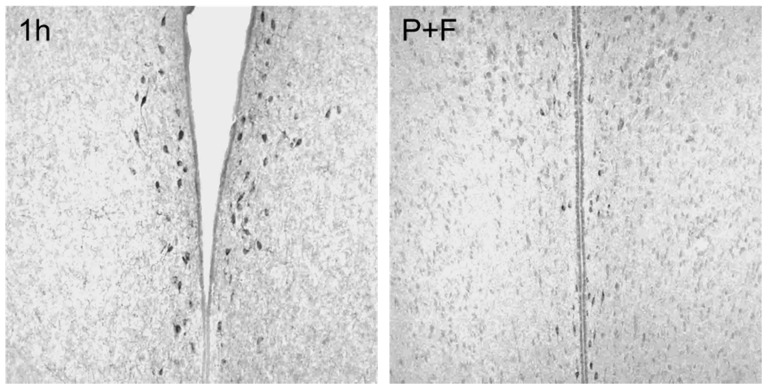
Somatostatin immunoreactivity in the medial periventricular nuclei of Syrian hamsters. Comparison between 1 h manual sleep deprivation (1 h, group 3a) and sexual stimulation with pheromone and females (P+F, group 1). Magnification: 50×.

**Figure A14:**
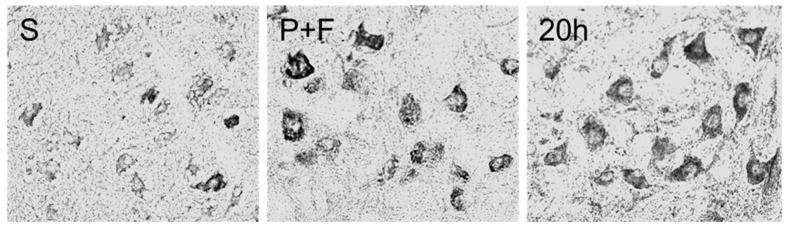
Dopamine β-hydroxylase immunoreactivity in area 7 of the brainstem of Syrian hamsters. Comparison between sleep (S, group 5), sexual stimulation with pheromone and females (P+F, group 1), and 20 h manual sleep deprivation (20 h, group 3c). Magnification: 200×.

**Figure A15:**
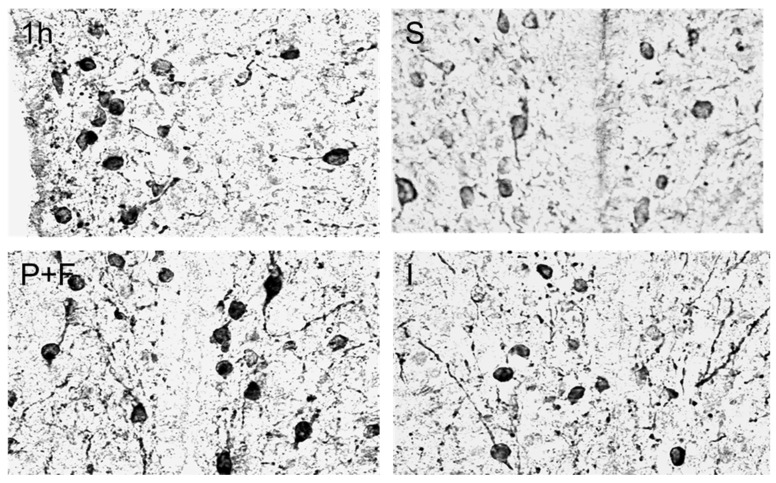
Tyrosine-hydroxylase immunoreactivity in the infundibular nucleus of Syrian hamsters. Comparison between 1 h manual sleep deprivation (1 h, group 3a), sleep (S, group 5), sexual stimulation with pheromone and females (P+F, group 1) and immobilization stress (I, group 4). Magnification: 200×.

In addition to the example pictures shown above in Figure 1, we provide 15 appendix Figure A1, Figure A2, Figure A3, Figure A4, Figure A5, Figure A6, Figure A7, Figure A8, Figure A9, Figure A10, Figure A11, Figure A12, Figure A13, Figure A14 and Figure A15 in the Appendix A which show micrographs specifically demonstrating the significant differences in the immunoreactivity as summarized in Table 2 above. In detail, those figures are composed of micrographs showing the immunoreactivity to particular neuromessengers in specific brain nuclei. Thereby, only images were selected which demonstrate significant differences in the immunoreactivity as summarized in Table 2 above.

## 4. Discussion

The study was intended to analyze specific effects of sexual arousal on neurotransmitter and neuropeptide systems as well as the time-keeping protein patterns in the SCN, in its magnocellular target neurons in the PVN and SON as well as in other related cerebral entities of Syrian hamsters, next to hormone levels in the blood and sexual steroid responsiveness as indicated by sexual steroid receptor density. To induce sexual arousal, the hamsters were stimulated with olfactory and optical cues during their main sleeping period. This type of stimulation was compared to sleep deprivation by mild manual handling and additionally compared to pheromone stimulation solely and to severe immobilization stress. Further, comparison with prolonged manual sleep deprivation and completed sleep periods was performed. The latter resulted in later zeitgeber time of sacrifice, however, we did not detect significant differences between scarification after terminating the sleep phase vs. that of hamsters after 1 h of sleep as described previously [29]. By mild manual handling, the (tamed) animals had to be repeatedly stimulated to prevent them from falling asleep for 1 h or longer during their main diurnal sleeping period, whereas severe stress caused high levels of alertness. As observed during the pre-tests, completely undisturbed golden hamsters could be woken up only by intense manual stimulation during this main sleeping period. The non-anthropogenic stimulation with species-specific pheromone, however, caused complete naturally based sleep deprivation with high levels of alertness, especially when supported by the presence of females. In line with these diversified behavioral effects, the reactivity pattern of the neurotransmitter and neuropeptide systems differed conspicuously among the three groups.

The behavioral changes of the assessed male hamsters as well as their markedly increased plasma testosterone levels suggest that the chosen experimental setting, i.e., the combination of an olfactory aphrodisin stimulation in the presence of females, was indeed suitable to induce sexual arousal. Steroids act largely permissive on male sexual behavior through interconnected limbic nuclei including the medial amygdala and the MPOA [4]. Indeed, the high concentration of blood testosterone associated with the sexual stimulation setting was associated with the down-regulation of sexual steroid receptors in the PVN, most likely decreasing steroid responsiveness, but not in the MPOA in this study. The sexual steroids’ quantitative effect partially depends on co-circulating catecholamines in addition to the bioelectric state of testosterone binding cells of the limbic system [37]. Indeed, an increase in epinephrinergic brain stem activity was exemplarily demonstrated for the A7 neuron population as indicated by DBH levels in good concordance with this observation. Interestingly, however, intermediate-term sleep deprivation of about 20 h resulted in a similar increase. In total, a threefold increase in blood testosterone could be achieved by our sexual stimulation setting. In contrast, acute immobilization stress [38,39] and chronic stress [40] are known to be associated with drastic and enduring decreases in plasma testosterone levels. In line with this, we observed reduced concentrations of blood testosterone due to immobilization stress for 1 h not only in comparison to sexual stimulation but even compared to short-term sleep deprivation about 6 h. We conclude that the optical and olfactory challenge associated with the sexual stimulation setting led to a mode of arousal other than the aversive stress setting and that it might have been associated with increased sexual appetence. Considerably different immunoreactivity patterns resulting from pheromone stimulation alone might indicate that the visual contact to females may have been of importance for a sexual appetence-associated interpretation of the pheromone stimulus.

The occurrence of appetence behavior and the drastic increase in blood testosterone in the sexually stimulated animals as compared to immobilization stress and sleep deprivation for 6 h are most likely caused by differential alterations in the messenger systems of the circadian pacemaker in the SCN and its input regions as well as its output targets. The most prominent efferent projections from the SCN lead to the PVN [41], which is believed to play an important role in the regulation of sexual behavior [42]. The nonapeptide AVP expressed in both nuclei is suggested as a messenger of this axis. AVP is believed to facilitate sexual behavior [43,44] with the medial amygdaloid nucleus and the hippocampus as prominent sites of behavioral control [43]. We observed decreases of immunoreactivity in the SCN compared to short-term manual sleep deprivation for 1 h associated with increased levels in the PVN compared to sleeping animals. Stress, particularly repeated stress, may increase AVP plasma levels due to activation of AVP synthesis in parvocellular corticotropin-releasing hormone-producing neurons of the PVN [45,46]. The AVP reaction pattern in the SCN due to acute immobilization stress was virtually identical to sexual stimulation, suggesting that this reaction may be conserved in rewarding and aversive arousal. However, immobilization stress also led to reduced AVP in SON compared to short-term manual sleep deprivation for 1 h as well as to increased AVP levels in blood compared to manual sleep deprivation for 6 h and 20 h.

In sharp contrast to AVP, both VIP and GRP seemed to be more pronouncedly influenced by their previously described diurnal rhythmic release in SCN neurons [32] than by the mode of external stimuli. In the described experimental setting, VIP was slightly but significantly increased by pheromone stimulation alone compared to combined stimulation with pheromone and the abundance of females as well as to sleep deprivation for 6 h with ZT 05.00 for sacrifice. GRP was more pronouncedly influenced by the duration than by the mode of sleep deprivation, with 20 h leading to a significant decrease in immunoreactivity in SCN compared to short-term manual sleep deprivation of 1 h or sleep ad libitum. The observed increase in GRP in the SCN of sleeping hamsters matches previous results [29] and indeed, VIP is known to be more important for peripheral reactions during sexual arousal [12,47,48], while the central nervous VIP release, e.g., in the hypothalamic median eminence, is known to be triggered by chronic but not by acute stress [49]. GRP seems to be more relevant for the type of sexual preferences in mice than for the sexual reaction itself [13]. Stress responses related to GRP were studied more extensively in the peripheral nervous system, e.g., depletions of GRP-containing vesicles in gastric nerve fibers caused by stress [50].

The neurotransmitter GABA is involved in neural processes inhibiting sexual activity at various central nervous sites [51,52], while GABA-antagonists facilitate mating behavior [53]. Partially, the negative GABA effect is influenced by olfactory stimuli [54]. However, prolonged but not acute stress is known to be associated with increased quantities of GABA in the hypothalamus of rats [55,56]. GAD-65/67 immunoreactivity in SCN neurons, however, was not affected by our sexual arousal setting, neither by the mode nor by the duration of sleep deprivation.

The general influence of intermediate-term manual sleep deprivation for 20 h compared to sleep on the immunoreactivity of the time-keeping proteins Clock, Per 1, Per 2 and Cry 2 [57,58,59,60,61] was recorded in SCN, PVN, in SON. Interestingly, an increase in Clock and Per 2 in SCN of the hamsters deprived of sleep compared to sleeping hamsters remained the only observed significant effects, most likely as compensatory phenomena as suggested before [57,58]. The increased Per 1 activity in the 20 h manual sleep deprivation group is well in line with the previously described Per 1 induction by serotonergic activity [62].

SP in the mesencephalic central grey can facilitate sexual activity in rats [63]. In afferent fibers to the SCN, SP immunoreactivity was drastically reduced after our sexual stimulation experiment. The SP reactivity to stress was reported to differ among various brain nuclei: decreased SP immunoreactivity was found in the septum, striatum and hippocampus of rats in response to acute stress [64], an increase in SP was observed in hypothalamic sites after exposure to prolonged stress for 24 h [65]. In spite of those previous observations on acute stress [64], no significant changes in afferent fibers projecting to the SCN were recorded due to immobilization stress for 1 h.

NPY predominantly shows inhibitory effects on sexual behavior [66,67]. In rats, the NPY response to repeated aversive stress is diversified and depends on the exact mode of the stimuli applied [67,68,69]. In the experimental setting as presented here, NPY immunoreactivity in afferent fibers to the SCN was decreased after pheromone-only stimulation and after immobilization stress compared to manual sleep deprivation for 1 h, while this phenomenon could not be significantly demonstrated for the sexual stimulation setting. At least with regard to this neurotransmitter, the solitary pheromone stimulation resulted in a reaction more similar to aversive stress than to sexual appetence.

Both ME and 5-HT are known to mediate inhibitory effects on sexual activity [8,10] and were, accordingly, measured in lower quantities in afferent fibers to the SCN after sexual stimulation compared to manual sleep deprivation for 1 h. The same applies to SOM immunoreactivity in PVNM neurons [9] which are known to project to the SCN [17]. In addition, in comparison to the sleeping animals, both intermediate-term manual sleep deprivation and short-term manual sleep deprivation were associated with increased 5-HT levels in afferent fibers in the SCN, a finding which is in line with previous results [70,71]. In the dorsal raphe nucleus, however, this change was not reproducible with decreased 5-HT immunoreactivity only after sole pheromone stimulation compared to manual sleep deprivation for 1 h. Since the dorsal raphe nucleus is influenced by epinephrinergic afferences from the brain stem [20], DBH as the guiding enzyme for the neurotransmitter epinephrine was exemplarily assessed in the A7 neuron population of the brain stem. The differentiated DBH reaction pattern in the A7 neuron population with significantly decreased immunoreactivity in sleeping hamsters compared to hamsters exposed to prolongated manual sleep deprivation for 20 h as well as to sexually stimulated hamsters, however, were neither directly nor reciprocally reflected by the 5-HT immunoreactivity on DRN level, suggesting a stronger dominance by other influences. However, it may reflect the known stimulating effects of the brainstem epinephrine on arousal [21].

Dopamine is a widely accepted neurotransmitter facilitating sexual activity [5]. In line with this, higher levels of tyrosine hydroxylase (TH), the guiding enzyme for dopamine synthesis, were measured in the infundibular nucleus (IN) of sexually stimulated animals compared to immobilized ones. Sleeping animals showed lower TH immunoreactivity compared to both sexually stimulated animals and animals deprived of sleep by gentle manual handling for a period of 1 h which is in good concordance with previous findings in other hypothalamic entities [72].

OXY widely facilitates acute sexual arousal [73,74,75], and, influenced by oxytocinergic neurons in the PVN, penile erections [76,77] and the orgasmic process [73,78]. The initial sexual encounter stimulates OXY secretion combined with the activation of synthesis in oxytocinergic neurons in hypothalamic nuclei [42]. However, our sexual arousal setting, similar to the 1 h manual sleep deprivation, showed only increased OXY levels in the SON compared to the group receiving pheromone stimulation solely, again suggesting different reactions due to pheromones alone compared to the combination of pheromones and females. No blood oxytocin was quantified, so it remains unknown whether the reaction in the sexually stimulated animals was due to low neuroendocrine secretion or due to a compensation of the secretion by intracellular de novo synthesis of OXY. Regarding OXY immunoreactivity in the PVN, there was even increased OXY immunoreactivity in hamsters manually deprived of sleep for 1 h as compared to this mode of sleep deprivation for 6 h and sleep ad libitum. On the other hand, the stimulation interval of 1 h might have been too short to detect alterations in the OXY synthesis and/or depletion from the magnocellular perikarya. In rats, acute exposure to immobilization stress was described to result in hypothalamic OXY synthesis and secretion mainly via the PVN [45] but not via the SON [79]. OXY immunoreactivity in both PVN and SON was, however, not significantly affected in our immobilization setting.

Increased testosterone blood levels suggested the achievement of the desired stimulating effect in our sexual stimulation group compared to the hamsters deprived of sleep by the aversive stimulus of immobilization stress [4]. The observed similar increase in the 6 h manual sleep deprivation group is an interesting phenomenon. In the PVN but not in the MPOA, compensatory down-regulation of sexual steroid receptors was observed in the sexually stimulated hamsters compared to the 1 h manual sleep deprivation group.

Since only one diagnostic method, i.e., semiquantitative immunohistochemistry, was applied for the quantification of each assessed parameter within the animals’ brains, the study implies an undeniable limitation; the non-stoichiometric immunoreactions may cause intraassay variations which limit the validity of results with smaller levels of significance. Accordingly, weak significance has to be considered as preliminary and hypothesis-forming in this way and future studies applying confirmatory methods, comprising, e.g., radioimmunoassays, quantitative Western blotting, high-pressure liquid chromatography (HPLC) or in situ hybridization targeting the messenger RNA of protein messengers, are highly desirable.

The fact that correction for multiple testing was not included in the statistical approach is another limitation of the study. Otherwise, a considerably higher number of animals would have been needed to show small differences in immunoreactivity. However, it cannot be excluded that some of the observed minor differences were in this way just by chance. This is another reason why some of the results should be considered as hypothesis-forming only.

As another limitation of the study, we did not discriminate potential responders and non-responders towards sleep deprivation. If there were non-responders among the animals, the respective impact on the assessed neuromessengers will most likely have affected the standard deviations of the measured results and contributed to partly weak significances for the recorded effects. However, since the Syrian hamsters used in our investigation were litter mates of an inbred lineage, this aspect should be of minor importance.

## 5. Conclusions

In the present investigation, different regimens of sleep deprivation were tested. All of them successfully prevent sleep during the main diurnal sleep phase. However, stress-induced and, somewhat surprisingly, pheromone-and-female-induced sleep prevention were much more effective than manual handling in the tamed golden hamsters. In line with this, the different neuromodulator systems displayed rather diversified reactivity patterns among the experimental groups. It is worthwhile to elucidate the potential neuronal mechanism, how the experimental settings might have affected the hypothalamic timing system responsible for the alterations in sleeping behavior.

Gentle manual handling in tamed animals may not be a strong waking signal; the animals are used to tactile stimuli which are perceived via fast adapting mechanoreceptors [80]. Sleep deprivation for more than 1 h was only possible by frequently repeated manual stimulations, and significant alterations in the neuromodulator systems of the hypothalamic timing control nuclei were not observed until 20 h of such permanent handling as compared to animals manually deprived of sleep for 6 h and animals that were allowed to sleep at libitum during their natural diurnal period of sleep.

Severe stress caused by our drastic type of immobilization directly affects the entire central nervous sympathetic control system including the amygdala via the locus coeruleus nuclei [81]. The latter is abundantly interconnected with the magnocellular hypothalamic nuclei, in particular with the PVN. The PVN is strongly interconnected with the SCN and, accordingly, influences the circadian clock and the timing system of sleep. The significant decrease in AVP and NPY after stress stimulation may be based on this input to the SCN. Furthermore, the PVN is the superior control entity for the sympathetic output via the neuronal (paravertebral sympathetic truncus) and humoral axis (AVP and ACTH) [82]. In line with these facts, we observed a decrease of AVP in the SON in the magnocellular system, resulting in an increase in blood. Unfortunately, the corticotropin-releasing factor-(CRF-) reactivity was not analyzed, because CRF was found to stimulate the synthesis of the sleep inhibiting peptide orexin [83].

The architecture of the combined neuronal and humoral pathway conveying olfactory stimuli to the testosterone secreting Leydig cells includes the terminal nerve, the olfactory tubercles and the preoptic nuclei as neural components as well as GnRH (gonadotropin releasing hormone) and LH (luteinizing hormone) as humoral components [84]. The strong increase in testosterone in the blood of golden hamsters stimulated by pheromone in the abundance of females is mediated via this axis. The increased testosterone levels can directly act on androgen receptors in the SCN [85] or in the medial preoptic nucleus [86]. The latter is interconnected with the PVN as well as with the lateral septum and with the amygdala. In the PVN, even a compensatory down-regulation of sexual steroid receptors was observed in our study. The amygdala and the septum, which are involved in emotional (and possibly olfactory) control, are connected with the PVN, the SON, and the SCN [87]. By activation of this axis, the observed alterations of neuromessenger immunoreactivity in the assessed hypothalamic nuclei may have been mediated or at least affected.

Generally, several reactivity patterns of neuromodulators behave similarly when comparing stress, pheromone-stimulation and combined pheromone-and-female-based sexual stimulation in Syrian hamsters, i.e., AVP in the SCN comparing sexual stimulation and immobilization, NP-Y in the SCN as well as AVP in the SON comparing pheromone-only stimulation and immobilization. These concordant patterns may be necessary to alter the sleep control via the hypothalamic melanin-concentrating hormone (MCH) and the orexin system which express strong sleep-promoting or sleep-preventing actions, respectively [88,89]., mediated by the axis in the hypothalamus and to facilitate the peripheral activity shown during both stress and pheromone/sexual stimulation.

## Figures and Tables

**Table 1:** List of antisera used for immunostaining. 5-HT = serotonin (5-hydroxy-tryptamin), AR = androgen receptor, AVP = arginine-vasopressin, Cry = cryptochrome, DBH = dopamine beta-hydroxylase (the guiding enzyme of norepinephrine synthesis), GAD-65/67 = glutamic acid decarboxylase (the guiding enzyme of GABA synthesis), GRP = gastrin-releasing peptide, ME = met-enkephalin, NPY = neuropeptide Y, OR = estrogen receptor, OXY = oxytocin, PER = Period, SOM = somatostatin, SP = substance P, TH = tyrosine hydroxylase (the guiding enzyme of dopamine synthesis), VIP = vasoactive intestinal polypeptide.

Antibody against	Working Dilution	Producer
AVP	1:1000	Own production [29]
VIP	1:1000	Sigma product, product number: V3508
GRP	1:500	Affinity Res. Prod., Exeter, UK
GAD-65/67	1:1000	Biotrend Chemikalien GmbH, Köln, Germany
5-HT	1:1000	Diasorin, Stillwater, MN, USA
SP	1:1000	Chemicon International, Temecula, CA, USA
NPY	1:1000	Affinity Res. Prod., Exeter, UK
ME	1:1000	Chemicon International, Temecula, CA, USA
OXY	1:1000	Own production [32]
SOM	1:1000	Chemicon International, Temecula, CA, USA
AR	1:50	Novocastra Laboratories, Newcastle, UK
OR	1:50	Novocastra Laboratories, Newcastle, UK
TH	1:1000	Chemicon International, Temecula, CA, USA
DBH	1:1000	Chemicon International, Temecula, CA, USA
Clock	1:60	Santa Cruz Biotechnology, Inc., Dallas, TX, USA
Per 1	1:60	Santa Cruz Biotechnology, Inc., Dallas, TX, USA
Per 2	1:60	Alpha Diagnostics Ltd., Reinach, Switzerland
Cry 2	1:60	Alpha Diagnostics Ltd., Reinach, Switzerland

**Table 2:** Effects of sleep deprivation caused by sexual arousal (group 1), pheromone-only based stimulation (group 2), gentle manual handling for 1 h (group 3a), 6 h (group 3b), and 20 h (group 3c), respectively, immobilization stress (group 4) versus a control group of hamsters sleeping ad libitum (group 5) on neuropeptides, time-keeping proteins, steroid receptors, guiding enzymes for the synthesis of neurotransmitters as well as the neurotransmitter serotonin and the steroid testosterone in sexually naïve male Syrian hamsters. 5-HT = serotonin (5-hydroxy-tryptophan), AR = androgen receptor, AVP = arginine-vasopressin, Cry = cryptochrome, DBH = dopamine beta-hydroxylase (the guiding enzyme of norepinephrine synthesis), GAD-65/67 = glutamate dehydrogenase (the guiding enzyme of GABA synthesis), GRP = gastrin-releasing peptide, ME = met-enkephalin, NPY = neuropeptide Y, OR = estrogen receptor, OXY = oxytocin, PER = period, pi = pixels, SOM = somatostatin, SP = substance P, TH = tyrosine hydroxylase (the guiding enzyme of dopamine synthesis),VIP = vasoactive intestinal polypeptide. ↓ = *p* < 0.05 for decrease compared to at least one other experimental group, ↑ = *p* < 0.05 for decrease compared to at least one other experimental group. A7 = A7 neuron population of the brain stem, DRN = dorsal raphe nucleus, IN = infundibular nucleus, MPOA = medial pre-optic area, MPVN = medial periventricular nucleus, PVN = paraventricular nucleus, SCN = suprachiasmatic nucleus, SON = supraoptic nucleus. ZT = zeitgeber time. - = data missing. Data are provided as mean values ± standard deviation.

Parameter	Entity	Group 1: 1 h Exposit. to Pheromones and Females, ZT 01.00,	Group 2: 1 h Exposit. to Pheromones, ZT 01.00,	Group 3a: 1 h Manual Sleep Deprivation, ZT 01.00,	Group 3b: 6 h Manual Sleep Deprivation, ZT 05.00,	Group 3c: 20 h Manual Sleep Deprivation, ZT 05.00,	Group 4: 1 h Exposit. to Immobiliza-tion Stress, ZT 01.00,	Group 5: Sleep Control: Sleeping Animals, ZT 05.00,	*p* (Significance over All Compared Groups)
AVP ^1^	SCN	0.45% (±0.22%) ↓	1.39% (±0.54%)	5.59% (±2.32%) ↑	0.67% (±0.17%)	1.62% (±0.39%)	0.62% (±0.36%) ↓	0.36% (±0.47%) ↓	0.0002
VIP ^2^	SCN	0.02% (±0.02%) ↓	0.30% (±0.11%) ↑	0.14% (±0.17%)	0.04% (±0.05%) ↓	0.09% (±0.07%)	0.15% (±0.04%)	0.43% (±0.33%)	0.0027
GRP ^3^	SCN	1.22% (±0.93%)	1.19% (±1.15%)	7.52% (±5.94%) ↑	1.37% (±0.41%)	0.06% (±0.02%) ↓	1.22% (±0.84%)	2.36% (±1.22%) ↑	0.0115
GAD65/67	SCN	7.26% (±2.94%)	7.64% (±4.77%)	7.64% (±2.43%)	12.15% (±3.30%)	9.37% (±4.48%)	4.05% (±1.94%)	14.35% (±7.89%)	0.0572
5-HT ^4^	SCN	481.60 pi (±155.91 pi) ↓	-	1625.20 pi. (±155.91 pi) ↑	520.00 pi. (±96.75 pi.)	959.40 pi. (±237.6 pi) ↑	-	292.75 pi. (±59.83 pi) ↓	0.0004
SP ^5^	SCN	0.04% (±0.01%) ↓	0.73% (±0.14%)	1.58% (±0.49%) ↑	-	-	0.63% (±0.25%)	-	0.0039
NPY ^6^	SCN	11.67% (±3.63%)	8.52% (±7.79%) ↓	28.67% (±4.95%) ↑	-	-	7.79% (±3.49%) ↓	-	0.0087
ME ^5^	SCN	0.38% (±0.15%) ↓	-	2.85% (±0.4%) ↑	-	-	-	-	0.0159
Clock ^7^	SCN	-	-	-	-	10.47% (±1.02%) ↑	-	8.13% (±0.81%) ↓	0.0286
Per 1	SCN	-	-	-	-	6.61% (±0.50%)	-	4.83% (±0.71%)	0.571
Per 2 ^7^	SCN	-	-	-	-	5.09% (±0.64%) ↑	-	3,92% (±0.15%) ↓	0.0286
Cry 2	SCN	-	-	-	-	6.85% (±1.76%)	-	6.61% (±0.58%)	>0.9999
AVP ^8^	PVN	7.01% (±0.9%) ↑	8.35% (±1.35%)	11.64% (±8.96%)	6.22% (±2.10%)	4.20% (±0.79%)	6.06% (±2.52%)	3.93% (±1.22%) ↓	0.0098
OXY ^9^	PVN	9.97% (±1.75%)	4.81% (±1.75%)	13.04% (±2.93%) ↑	3.31% (±3.36%) ↓	2.96% (±1.27%)	4.54% (±2.20%)	2.04% (±0.36%) ↓	0.0022
AR ^5^	PVN	0.53% (±0.39%) ↓	-	1.91% (±0.82%) ↑	-	-	-	-	0.0317
OR ^5^	PVN	0.70% (±0.37%) ↓	-	2.17% (±0.78%) ↑	-	-	-	-	0.0159
Clock	PVN	-	-	-	-	6.13% (±0.24%)	-	4.13% (±0.83%)	0.0571
Per 1	PVN	-	-	-	-	5.58% (±0.84%)	-	5.77% (±0.59%)	0.8571
Per 2	PVN	-	-	-	-	2.77% (±0.82%)	-	3.16% (±0.28%) *	>0.9999
Cry 2	PVN	-	-	-	-	4.17% (±0.80%)	-	4.59% (±1.22%)	0.6286
AVP ^10^	SON	22.56% (±3.26%)	10.18% (±3.09%) ↓	66.60% (±4.53%) ↑	46.23% (±3.20%) ↑	47.19% (±3.46%) ↑	6.85% (±1.54%) ↓	36.03% (±7.75%)	<0.0001
OXY ^11^	SON	17.30% (±4.05%) ↑	3.97% (±1.21%) ↓	15.39% (±1.89%) ↑	-	-	6.20% (±0.82%)	-	0.0018
Clock	SON	-	-	-	-	6.17% (±0.72%)	-	3.03% (±0.55%)	0.0571
Per 1	SON	-	-	-	-	5.37% (±0.64%)	-	3.66% (±1.01%)	0.1143
Per 2	SON	-	-	-	-	2.86% (±0.58%)	-	3.22% (±0.57%)	0.4000
Cry 2	SON	-	-	-	-	4.27% (±0.70%)	-	4.24% (±0.51%)	0.8571
AR	MPOA	0.55% (±0.65%)	-	12.29% (±8.2%)	9.62% (±1.50%)	7.74% (n.e.) *	-	12.38% (±4.89%)	0.0493
OR	MPOA	12.65% (±8.67%)	-	21.35% (±4.44%)	22.21% (±3.30%)	16.55% (n.e.) *	-	23.32% (±13.75%)	0.4540
SOM ^5^	MPVN	2.45% (±0.24%) ↓	-	8.63% (±2.67%) ↑	-	-	-	-	0.0159
TH ^12^	IN	9.15% (±1.52%) ↑	6.83% (±4.99%)	7.84% (±0.41%) ↑	3.67% (±0.69%)	4.96% (±0.45%)	3.37% (±2.01%) ↓	2.30% (±0.25%) ↓	0.0005
5-HT ^13^	DRN	7.22% (±4.8%)	2.16% (±0.96%) ↓	11.45% (±2.19%) ↑	11.33% (±4.06%)	10.20% (±2.36%)	3.56% (±0.89%)	8.47% (±2.62%)	0.0077
DBH ^14^	A7	5.04% (±0.82%) ↑	-	2.83% (±1.04%)	2.35% (±0.65%)	5.33% (±1.20%) ↑	-	2.17% (±0.55%) ↓	0.0029
AVP ^15^	Blood	5.84 pg/mL (±0.86 pg/mL)	18.00 pg/mL (±25.51 pg/mL)	3.94 pg/mL (±1.56 pg/mL)	3.00 pg/mL (±0.79 pg/mL) ↓	4.00 pg/mL (±0.96 pg/mL)	111.0 pg/mL (±87.7 pg/mL) ↑	3.34 pg/mL (±2.14 pg/mL) ↓	0.0153
Testo-sterone ^16^	Blood	9.42 ng/mL (±1.73 ng/mL) ↑	4.12 ng/mL (±1.20 ng/mL)	3.26 ng/mL (±0.72 ng/mL)	8.25 ng/mL (±3.61 ng/mL) ↑	4.38 ng/mL (±3.72 ng/mL)	1.63 ng/mL (±0.46 ng/mL) ↓	5.40 ng/mL (±2.62 ng/mL)	0.0017

* Slides from less than three animals included. n.e. = not estimable. ^1^ 1 h manual sleep deprivation (ZT 01.00) increased compared to sleeping animals (ZT 05.00; *p* < 0.01), to 1 h exposition to pheromones and females (ZT 01.00; *p* < 0.01), and to 1 h immobilization stress (ZT 01.00; *p* < 0.05); ^2^ 1 h exposition to pheromones (ZT 01.00) increased compared to 6 h sleep deprivation (ZT 05.00; *p* < 0.05) and to 1 h exposition to pheromones and females (ZT 01.00; *p* < 0.05); ^3^ 20 h manual sleep deprivation (ZT 05.00) reduced compared to sleep (ZT 05.00; *p* < 0.05) and to 1 h sleep deprivation (ZT 01.00; *p* < 0.01); ^4^ sleep (ZT 05.00) reduced compared to 20 h manual sleep deprivation (ZT 05.00; *p* < 0.05) and to 1 h manual sleep deprivation (ZT 01.00; *p* < 0.001), the latter, in addition, increased compared to 1 h exposition to pheromones and females (ZT 01.00; *p* < 0.05); ^5^ 1 h manual sleep deprivation (ZT 01.00) increased compared to 1 h exposition to pheromones and females (ZT 01.00; *p* < 0.01); ^6^ 1 h manual sleep deprivation (ZT 01.00) increased compared to 1 h exposition to pheromones (ZT 01.00; *p* < 0.05) and to 1 h immobilization stress (ZT 01.00; *p* < 0.05); ^7^ sleep (ZT 05.00) reduced compared to 20 h manual sleep deprivation (ZT 05.00; *p* < 0.05); ^8^ sleep (ZT 05.00) reduced compared to 1 h exposition to pheromones and females (ZT 01.00; *p* < 0.05); ^9^ 1 h manual sleep deprivation (ZT 01.00) increased compared to 6 h manual sleep deprivation (ZT 05.00; *p* < 0.05) and to sleep (ZT 05.00; *p* < 0.05); ^10^ 1 h immobilization stress (ZT 01.00) reduced compared to 20 h manual sleep deprivation (ZT 05.00; *p* < 0.05), to 6 h manual sleep deprivation (ZT 05.00; *p* < 0.05), and to 1 h manual sleep deprivation (ZT 01.00; *p* < 0.05), the latter, in addition, increased compared to 1 h exposition to pheromones (ZT 01.00; *p* < 0.05); ^11^ 1 h exposition to pheromones (ZT 01.00) reduced compared to 1 h exposition to pheromones and females (ZT 01.00; *p* < 0.01) and to 1 h manual sleep deprivation (ZT 01.00; *p* < 0.05); ^12^ sleep (ZT 05.00) reduced compared to 1 h manual sleep deprivation (ZT 01.00; *p* < 0.05) and to 1 h exposition to pheromones and females (ZT 01.00; *p* < 0.01), the latter, in addition, increased compared to 1 h immobilization stress (ZT 01.00; *p* < 0.05); ^13^ 1 h exposition to pheromones (ZT 01.00) reduced compared to 1 h manual sleep deprivation (ZT 01.00; *p* < 0.05), ^14^ sleep (ZT 05.00) reduced compared to 20 h manual sleep deprivation (ZT 05.00; *p* < 0.05) and to 1 h exposition to pheromones and females (ZT 01.00; *p* < 0.05); ^15^ immobilization stress (ZT 01.00) increased compared to sleep (ZT 05.00; *p* < 0.05) and to 6 h manual sleep deprivation (ZT 05.00; *p* < 0.05); ^16^ immobilization stress (ZT 01.00) reduced compared to 1 h exposition to pheromones and females (ZT 01.00; *p* < 0.01) and to 6 h manual sleep deprivation (ZT 05.00; *p* < 0.01).

**Table 3:** Neuromessengers with relative increase or decrease compared to at least one other experimental group for different entities. 5-HT = serotonin (5-hydroxy-tryptophan), AR = androgen receptor, AVP = arginine-vasopressin, DBH = dopamine beta-hydroxylase (the guiding enzyme of norepinephrine synthesis), GRP = gastrin-releasing peptide, ME = met-enkephalin, NP-Y = neuropeptide Y, OR = estrogen receptor, OXY = oxytocin, PER = Period, SOM = somatostatin, SP = substance P, TH = tyrosine hydroxylase (the guiding enzyme of dopamine synthesis), VIP = vasoactive intestinal polypeptide. A7 = A7 neuron population of the brain stem, DRN = dorsal raphe nucleus, IN = infundibular nucleus, MPVN = medial periventricular nucleus, PVN = paraventricular nucleus, SCN = suprachiasmatic nucleus, SON = supraoptic nucleus. ZT = zeitgeber time.

Experimental Group	Sacrifice Time (ZT)	Relative Increase Compared to at Least One Other Group	Relative Decrease Compared to at Least One Other Group
Intermediate-term sleep deprivation (20 h)	05.00	SCN: 5-HT, Clock, Per 2 SON: AVP A7: DBH	SCN: GRP
Short-term sleep deprivation (6 h)	05.00	SON: AVP Blood: testosterone	SCN: VIP PVN: OXY Blood: AVP
Short-term sleep deprivation (1 h)	01.00	SCN: AVP, GRP, 5-HT, SP, NP-Y, ME PVN: OXY, AR, OR SON: AVP, OXY MPVN: SOM IN: TH DRN: 5-HT	
Sleep	05.00	SCN: GRP	SCN: AVP, 5′-HT, Clock, Per 2 PVN: AVP, OXY IN: TH A7: DBH blood: AVP
Exposure to pheromone (1 h)	01.00	SCN: VIP	SCN: NP-Y SON: AVP, OXY DRN: 5-HT
Exposure to pheromone and females (1 h)	01.00	PVN: AVP SON: OXY IN: TH A7: DBH Blood: testosterone	SCN: AVP, VIP, 5-HT, SP, ME PVN: AR, OR MPVN: SOM
Immobilization stress (1 h)	01.00	Blood: AVP	SCN: AVP, NP-Y SON: AVP IN: TH Blood: testosterone

## Data Availability

All relevant data are provided in the manuscript. Raw data can be provided upon reasonable request.
